# From Blue to Pink
*Presidential Address: Delivered on 10th October, 1956.


**Published:** 1957-04

**Authors:** E. G. Bradbeer

**Affiliations:** Consultant Anaesthetist, United Bristol Hospitals.


### FROM BLUE TO PINK*

BY

E. G. BRADBEER, M.R.C.S., L.R.C.P., F.F.A.R.C.S., D.A.
*Consultant Anaesthetist, United Bristol Hospitals.*

My first duty is to thank you for the honour you have conferred on me in elect[illegible] and installing me as your President. I am aware that there are a number of [illegible] contemporary colleagues who would fill this high office much more ably than I, an[illegible] feel therefore, that my adornment with this handsome Badge of Office is in part[illegible] all events, a compliment to my specialty, and so, unworthy as I am, I felt c[illegible] strained to accept nomination on behalf of my anaesthetist friends. Talking [illegible] unworthiness reminds one of the story of the advice which was being given to [illegible] young man: "Never tell your girl friend you are unworthy; let it come as a surpris[illegible]

Two anaesthetists have previously held the Office of President of this Soci[illegible] The first was our dear old colleague, the late Dr. A. L. Flemming, who was Sen[illegible] Honorary Anaesthetist at the Bristol Royal Infirmary, and for whom I had the hon[illegible] and privilege of deputizing very frequently during his last few years on the St[illegible] The second was our old friend, Leonard Moore, who now lives in Cornwall, enjoy[illegible] a well-earned retirement. They were both my senior colleagues, together with [illegible] late Stewart Stock, when I was appointed Honorary Assistant Anaesthetist to [illegible] Bristol Royal Infirmary in 1925.

PROGRESS IN ANAESTHESIA

The choice of subjects on these occasions is left to the President, and it may [illegible] medical or non-medical. It seemed to me that, in my case, the subject chose its[illegible] The progress of Anaesthesia has been so great during the past 30 years, and I h[illegible] been privileged to have been actively engaged in anaesthesia during this time, [illegible] exclusively so during the major part of it, that I thought a general survey of [illegible] changing concept of anaesthesia over this period might be of interest.

I do not propose to tire you with detailed descriptions of drugs, techniques [illegible] apparatus, but to give you my impressions of the various changes which have ta[illegible] place in the specialty since the end of the First World War.

Those of you who may not have entered an operating theatre for 30 years may [illegible] interested in more recent developments, and those who are more recently quali[illegible] may feel stunned and somewhat aghast at the old rough-and-ready methods wh[illegible] were in vogue at that time. I regret that I cannot claim to have invented or [illegible] covered any new method or drug, yet I have been privileged, with my colleagues, [illegible] take part in the introduction in this region of all the new drugs and techniq[illegible] during this period. Moreover, some guiding hand, from a sphere even higher t[illegible] that of the surgeon, led me to avoid and discourage those methods and drugs wh[illegible] subsequently proved to be of doubtful value.

In order to obtain some sort of perspective, perhaps we should very briefly scan [illegible] past century, for anaesthesia as we know it is of very recent origin. By present-[illegible] standards of speed, it seems strange that it took so long for the known pain-reliev[illegible] effects of nitrous oxide and ether to be actually applied for the relief of pain dur[illegible] surgical operations.

Priestley, who in 1771 had discovered oxygen, described in 1777 the gas Nitro[illegible] Oxide, and the properties and potentialities of this gas in respect of produc[illegible] unconsciousness were described by Humphry Davy in 1799, whilst working here [illegible]

* Presidential address: delivered on 10th October, 1956.

Bristol at the Hotwells Pneumatic Institute, and he also suggested that the gas might be used in relieving pain of surgical operations. In 1818, Faraday discovered the narcotic action of ether, but still no application to surgery. Henry Hill Hickman, the Shropshire lad from Ludlow, published his paper, "A letter on Suspended Animation" in 1824, following his operations on animals which he rendered unconscious with Carbon Dioxide. I must apologize to you and to the memory of these distinguished men for so briefly mentioning their achievements, but, as I said before, I wished to draw your attention to the time interval involved.

It was not until 1844, nearly half a century after Humphry Davy's observations, that the actual use of nitrous oxide as an anaesthetic was inaugurated by Horace Wells, the Connecticut dentist. The first experiment was carried out with Wells himself as the patient. I am sorry we cannot dwell on the thrilling story of this epoch-making period, but merely pause to mention the names of Morton and Jackson and Crawford Long and the use of ether in 1846, and the introduction of chloroform by James Young Simpson in 1847. Then there followed the immortal John Snow and his successor Joseph T. Clover.

From this period until the early 1920's, general anaesthesia for everyday routine use consisted essentially of chloroform, ether, nitrous oxide and ethyl chloride, either separately or in combination, administered either by dropping on to a gauze mask or by some apparatus such as Clover's bag, and later, by Junker's inhaler, Kelly's apparatus or Shipway's apparatus.

Thus, although there was a very long period with no practical application of any really improved method of anaesthesia, the groundwork of many of the recent advances took place, such as the invention of the hollow needle in the last century, endotracheal intubation by MacEwen in 1878, and spinal analgesia by Corning and by August Bier, with Bier himself being the first volunteer in 1899.

You may well ask, that having obtained a method of anaesthesia which had been more of less successful for seventy years, in what direction could improvement lie? To consider this we must remind ourselves of the basic requirements of an anaesthetic:

1. Comfort and well-being of the patient before and after operation, with the absence of psychological trauma.

2. Safety of the patient, not only of life, but safety from subsequent temporary or permanent complications.

3. Perfect operative field for the surgeon.

### MY EARLY DAYS

I would like to tell you of my own introduction to the practice of anaesthesia. On my first day as a surgical dresser, I was told to be in the anaesthetic room and to function as a "holder-down". The anaesthetist was a house-physician who had just been demobilized at the end of the First World War, and he considered himself quite an expert anaesthetist. His technique was to have four "holders-down", one for each arm and one for each leg, whilst he himself dealt with the head. He then wrapped a towel around the patient's face, leaving the mouth and nose at the bottom of the well thus formed. A gauze mask of the Schimmelbusch type was placed over the hole, and another folded towel draped over all. Disdaining the ether dropper in the cork, which he always removed, he then proceeded to pour half the eight-ounce bottle of ether on to the towel, and everbody was told to hold tight! When all struggling ceased the patient was ready for operation.

I was very frightened of anaesthetics for some time after this, although I was to learn that it was possible to induce anaesthesia in a less brutal way. It may be that the very horror of my introduction led me later to take an interest in anaesthesia in the hope that one could, perhaps, do something to alleviate the fear that seized patients at the prospect of being anaesthetized.

In those days, anaesthesia was a part-time hobby, and one earned one's living by other means, usually general practice. In the twenties, there were four anaesthetists at the Royal Infirmary, and the youngest of my three senior colleagues was over twe[illegible] years my senior. During my early years on the Staff, on the few occasions when [illegible] services were requested in private by a certain well-known surgeon, the follow[illegible] monologue was invariably recited: "Good morning, Mr. So-and-so, I am ver[illegible] sorry to say that Dr. Flemming is ill and Dr. Moore is away, and so I have broug[illegible] along Dr. Bradbeer." We did not have L-plates in those days, but I might w[illegible] have introduced them.

### EARLY ANAESTHETIC APPARATUS

We are accustomed nowadays to see elegant, stream-lined, chromium-pla[illegible] anaesthetic apparatus in the theatre, and the machines of the 'twenties would now app[illegible] to be very primitive. Many were home-made, and the bottles for the water-flo[illegible] meters and the chloroform and ether containers were usually Hornby's milk bott[illegible] Even so, newer machines tended to be bigger and better. Some of you will rememb[illegible] Stuart Stock, one of whose hobbies was wood and metal working, and who was [illegible] expert model-yacht builder. He decided to build the biggest and best anaesthe[illegible] machine yet, and when he had built the trolley and framework, he installed it in No[illegible] anaesthetic room. Now, I always think this is where he made his big mistake. On t[illegible] framework at the back, he put a large blank sheet of paper with the inscription "A[illegible] suggestions"! I do not think this is the time or place to record the proffered advi[illegible]

Another interesting machine made by a member of the surgical staff who w[illegible] interested in anaesthesia, was like a magnificent radio set, with lots of knobs. Its o[illegible] disadvantage was that whatever knobs were turned, the resultant mixture was unp[illegible] dictable: at various times not only gases and vapours issued from the delivery tu[illegible] but also liquid ether and liquid chloroform, and even water. It was rumoured that, [illegible] one occasion, a glass of George's Home Brewed issued forth.

One of the first dental gases I was asked to give in private was interesting from t[illegible] point of view of apparatus. It was an extremely respectable and old-established Clift[illegible] practice, and I expect I was asked because Dr. Flemming was ill and Dr. Moore [illegible] away. This practice had not adopted the new-fangled cylinders of nitrous oxide, [illegible] still made its own gas in the basement, storing it in the surgery in a gasometer. I ha[illegible] not seen one since, and I feel very privileged to have had the opportunity of using [illegible] The dentist, poor fellow, passed on soon after my visit!

Nitrous oxide is a very weak anaesthetic, and when used for major surgical wo[illegible] must be combined with some other drug, and in those days chloroform and eth[illegible] vapours were added, most of the machines being modelled on Boyle's apparatus. T[illegible] flow of gases in these machines was measured very roughly by a water-flow-meter, a[illegible] one had to rely on anaesthesia being produced by the chloroform and ether, as with t[illegible] open drop method. One striking difference, however, was that the patients under t[illegible] form of anaesthesia were a far better colour, sometimes referred to as "Bristol Pink[illegible] and they also finished up in far better fettle than under the open-drop method. O[illegible] was led to the conclusion that it was the higher percentage of oxygen which contr[illegible] buted to this result. It occurred to me, therefore, that if a Boyle's machine was n[illegible] available, all one needed was a cylinder of oxygen and a piece of rubber tubing to lea[illegible] gentle flow of oxygen under the gauze mask in the open drop method during the wh[illegible] of the operation. You may well smile at this as being very elementary and obvious, b[illegible] I can assure you, ladies and gentlemen, that this was not the reaction it produced in t[illegible] operating theatre in those days. The request for a cylinder of oxygen was a sig[illegible] which drew all eyes to the patient's face, the assumption being that his demise w[illegible] expected at any minute, if indeed, it had not already occurred; and so, in order to av[illegible] spreading alarm and despondency, one had when adopting this method to smugg[illegible] an oxygen cylinder into the theatre surreptitiously.

One other factor which contributed to the more satisfactory condition of the patie[illegible] when using the Boyle's apparatus was the more adequate depth of breathing, due to t[illegible] slight build-up of carbon dioxide in this semi-closed method.

There were three important developments during the latter part of the 'twenties. None were altogether new, but their adoption in routine practice was.

### BASAL NARCOTICS

The first concerned basal narcosis, pre-operative sedation. Many anaesthetists used morphia and atropine, but others omitted the morphia on account of the depression of respiration, particularly in association with chloroform anaesthesia.

One of the factors which stimulated the investigation and extension of pre-operative sedation was the relative failure of local and regional analgesia for the toxic thyroid. The earliest and most successful drug for this purpose was rectal paraldehyde. This was relatively safe, very effective and what was very important, did not depress the respiration. I said it was relatively safe, bearing in mind one fatality which came to my notice where ounces were administered in mistake for drachms. After hearing of this case I always wrote the word *drachm* in preference to using the rather unsatisfactory wiggly symbol.

The successful and safe use of paraldehyde in thyroidectomies with the advantages to the peace of mind of the patient soon led to its use as a routine pre-operative procedure in all branches of surgery, especially children. The chief objection was its very penetrating and rather unpleasent smell. Rectal avertin, known now as bromethol, was introduced about the same time, and was favoured by many in preference to paraldehyde. Its action was more certain, but it needed very accurate measurement of the patient's weight, colour-testing of each individual dose and correct temperature at the time of administration.

### INTRAVENOUS ANAESTHETICS

The first of the intravenous anaesthetics—Pernocton—came to us about 1928. It was stable in solution, and its effect in producing sleep was as immediate as with the later Evipan and Pentothal, but the depth was not so profound: that is to say, a painful stimulus would produce reflex movement. Its chief disadvantage, was its very long period of amnesia, as shown in the following case. The patient was an Indian lady; incidentally, she was one of the most gracious, cultured and kindliest women I ever met. The operation was hysterectomy and she was in a private ward at the B.R.I. Two hours after return to her ward she was talking quite rationally to her nurse and behaving quite normally. Two hours later the nurse looked into the room and found her out of bed, dressing and saying she was going home Now, on the following day the patient had no recollection whatever of this incident, and I am glad to say that no untoward complication arose.

The barbiturates, sodium amytal and nembutal were also used by the intravenous route. Then in 1931 came Evipan, a new barbiturate which produced a short period of anaesthesia deep enough to perform surgical operations without the use of any supplementary anaesthetic. In 1935, Pentothal (sodium thiopentone), similar in action, was introduced, and very soon became universally popular for short surgical procedures and for basal narcosis. It was also used for longer operations in the form of a drip or by intermittent injection, the disadvantage for this purpose being the profound respiratory depression and consequent anoxia, and so nitrous oxide and oxygen was usually administered as well, chiefly for the benefit of the richer oxygen content and slight carbon dioxide build-up.

Although Pentothal was introduced as a very safe form of anaesthesia, it has in fact, proved to be one of the most lethal. Some people claim that it has killed more people than chloroform ever did. It has been said that at Pearl Harbour more people died as the result of Pentothal administration than from enemy action! Whether this may be true or not, it is, of course, the old story; the drug is safe enough, its danger lies in unskilled administration. I should also mention amongst the disastrous complication of Penthotal, the inadvertent injection of the drug into an artery, and to a lesser extent, into the tissues.

### SPINAL ANALGESIA

Spinal analgesia, since its introduction at the end of the last century has had a chequered career, and its early progress was marked by a dismal series of disas[illegible] However, if its administration could be rendered safe, the advantages to the surg[illegible] particularly in abdominal work, were very great on account of the prof[illegible] relaxation of the abdominal muscles. Among the causes of the unsatisfactory resu[illegible] that time were: toxic solutions, absence of standardization of the drugs, inadeq[illegible] sterilization of the solutions, inadequate control of the spread of the solution in[illegible] theca, unwise selection of patients and inadequate aseptic precautions.

My own introduction to spinal analgesia was in 1928, when Dr. Pitkin, a surg[illegible] from a town in the Middle West of the United States, visited the Royal Infirmary [illegible] ing his world tour. He was anxious to do the best surgery, and, handicapped by [illegible] low standard of general anaesthesia available to him, had devoted a large amou[illegible] time and thought and experiment to rationalizing spinal analgesia and making it [illegible] As a result of his researches, he produced a solution which was distributed unde[illegible] name of Spinocain, and this, in conjuction with the correct method of administr[illegible] and choice of patient certainly proved to be a considerable advance. On the after[illegible] that Pitkin came to the Royal Infirmary, I was anaesthetizing for Rendle Short, [illegible] had two gall-bladder cases on his list. Dr. Pitkin was invited to administer his Spino[illegible] for these two cases and he also performed the operations, with complete succe[illegible] think Mr. Jackman will recall Pitkin's visit because he was operating in the next the[illegible] and invited Pitkin to give his patient a spinal for inguinal hernia, and in his own [illegible] inimitable way, explained that he had not planted this patient there on purpose, [illegible] that it just happened that he was on the list, and was in fact in the anaesthetic room[illegible] the snag was that he had just the worst degree of scoliosis it was possible to imag[illegible] However, Dr. Pitkin was a sportsman and accepted the challenge, but, unfortun[illegible] was unsuccessful in finding a way into the theca.

Following the stimulus given by Pitkin and others, spinal analgesia again bec[illegible] very popular, and where used with the correct technique and safeguards was as sa[illegible] any existing method of anaesthesia.

Spinocain was followed a few years later by Percain, which was introduced by [illegible] late Howard Jones, who also evolved a successful technique. The name was cha[illegible] to Nupercain owing to a disaster where a mistake was made due to the similarity o[illegible] words *percain* and *procain*.

Whereas a tragedy with general anaesthesia usually resulted in death, that [illegible] spinal analgesia more often resulted in some permanent paralytic condition, so tha[illegible] tragedy lived on to the detriment of the method. Most of these misfortunes, how[illegible] were the result of untrained or careless administration, often both, for there are [illegible] anaesthetists who have given thousands of spinal anaesthetics without a single [illegible] neurological complication. The most elementary careless mistake in the injection [illegible] the theca of the wrong solution, and it is terrifying to hear of the variety of substa[illegible] which have been injected into the thecal canal in error. Before the war, I was aske[illegible] one of the defence societies to give an opinion in a legal case in which the w[illegible] solution had been injected and the patient died. The defence was that it was [illegible] responsibility of the Theatre Sister to fill the spinal syringe, and I was asked to [illegible] that this was a usual custom. Now, I had always taught just the opposite and [illegible] always strongly held the view that the anaesthetist was solely responsible for [illegible] injection of the correct substance. Since I could not agree to appear in a court of [illegible] and give an opinion directly contrary to my usual practice and teaching I had resp[illegible] fully to decline.

There are, of course, many pitfalls in the spinal technique for the untrained [illegible] unwary. Another elementary one is the neglect of meticulous asepsis. During the [illegible] when many things were scarce, we were sometimes short of sterile gowns and glo[illegible] and my dictum was that if an operation was to be performed under spinal analg[illegible]

and there was only one sterile gown and one pair of sterile gloves, then it was far more important for the anaesthetist to wear them for the spinal procedure than for the surgeon to wear them for the operation! I am quite sure it was as important as that, and yet often one saw spinal anaesthetics being given with a peremptory wash-up without sterile gloves or gown.

To pass for a moment to the lighter side, some of you may remember a certain surgeon who practised in one of the Welsh valleys, whom we will call Mr. X, who periodically visited the Royal Infirmary to keep abreast of the times. Mr. X was very portly and had a deep, rumbling voice. One fine day, in our early spinal days, Mr. Jackman was operating on a patient for whom I had given a spinal anaesthetic, and one of our rituals was complete silence in the theatre on these occasions. Who should enter the theatre but Mr. X, and resting his abdomen on the operating table at Mr. Jackman's left elbow, as was his wont, he said in his deep, rumbling voice, "I say, Jackman, did you read about that post mortem where a chap died under spinal anaesthesia?" Everyone was goggle-eyed, but, still silent, pointed to the poor patient. Was Mr. X's face red? I have never seen so big a man shrivel and fade out of a room so rapidly.

Is anything new? I read in a journal a few weeks ago that radio was introduced in an operating theatre in Paris for the benefit if the patient. Mr. Jackman will remember that over twenty years ago we tried this and also let the patient smoke a cigarette during his operation. It was not an unqualified success, however, and we soon abandoned the innovation.

### ENDOTRACHEAL INTUBATION

The third important advance during the 'twenties was Magill's introduction, or re-introduction, of the wide-bore endotracheal tube. Previous to this, endotracheal insufflation anaesthesia had been used by means of apparatus such as that designed by Kelly and Shipway to blow a jet of warmed ether vapour through a narrow gum-elastic catheter. This blew a continuous jet of ether vapour, which impinged on one spot in the trachea, and if continued for one or two hours produced a severe tracheitis. Magill's problem was in the severe facial injuries following the First World War, where it was necessary to provide for to-and-fro breathing in the presence of excessive bleeding in the pharynx. The wide-bore endotracheal tube was the answer, as it simply prolonged the trachea to the mouth, and to-and-fro breathing took place through the tube, so that the pharynx could be completely sealed off with a gauze pack.

I am indebted to my old colleague, Angell James, who returned to Bristol in 1928 on appointment to the Staff of the E.N.T. Department at the B.R.I., for stimulating me to adopt Magill's wide-bore endotracheal technique for his nose and throat operations. This was not so simple as it might appear today. I had to acquire all the equipment myself, and this included a modification of the old Boyle's apparatus. As I have already told you, my three colleagues were all very senior, and did not altogether approve of this rather alarming procedure, and forecast the incidence of large numbers of cases of papilloma of the chords. However, I bought tubes, Magill's laryngoscope, set of endotracheal connexions and Magill's modification of Boyle's apparatus, and as Angell James will remember, for the next two or three years when he had a case at the B.R.I., such as laryngectomy, external ethmoidectomy or antral carcinoma, I had to bring all my own equipment to the hospital for this purpose. Now, of course, you will find a plentiful supply of this type of apparatus in every theatre. It soon became apparent that the smoothness of the anaesthesia obtained by the inhalation endotracheal technique with its perfect airway from trachea to mouth made it very suitable for many types of operation from thyroidectomy to abdominal cases, and also for cases where the prone or lateral positions were required.

So, the position at the outbreak of the second World War was that intravenous barbiturates, spinal analgesia and endotracheal anaesthesia had become normal everyday procedures. Closed circuit anaesthesia with cyclopropane was also becoming popular, and during the early part of the war, trichlorethylene (Trilene) was a introduced. The introduction of the latter was interesting. This chemical was be largely used in the commercial cleaning of clothes, and a number of cases were cropp up where the operators were found lying unconscious on the floor. Recovery alw appeared to be complete, which indicated complete reversibility, and so some bri lad thought this might be an additional drug for use in aneasthesia. The great advant of Trilene is its non-inflammability, particularly at the present time when diather is almost universally used for haemostasis.

### THE "RELAXANTS"

In my opinoin, one of the greatest advances in anaesthesia was the introductio 1942 of curare by Griffiths and Johnson of Montreal. Since then, drugs other t curare having a similar action have been introduced, and these are now usually cal "relaxants".

By the intravenous route, the action of these drugs is to produce a paralytic effect the voluntary muscles at the site of the nerve-muscle junction, which for the abdomi surgeon presents the same perfect operative field as spinal analgesia does, but wi out the risks inherent in invasion of the thecal canal. The anaesthesia may, indeed should be very light, for herein lies the great advantage of the techniq Previously profound relaxation of the abdominal muscles with general anaesthe could only be obtained by soaking the patient with ether and chloroform, but now o the lightest level of the third stage of anaesthesia is necessary, and some anaesthe consider that only the second stage is required, providing complete flaccidity w the relaxant is assured.

We were fortunate here in Bristol in being early in the field in the use of cu before the war ended, because supplies of curare in this country were extrem limited, and a small amount was allowed to the few hospitals which commenced usi it in the early stages. At that time, the only literature consisted of Griffiths' origi articles, and we had to feel our way cautiously. Now, of course, an incredible mas literature has been published on the theory and mode of action of the various types relaxant. I should point out that in producing this flaccid condition of the abdomi muscles, the respiratory muscles also share in the paralytic phenomenon, and so it i *sine qua non*, that adequate artificial respiration must be maintained as long as t paralysis continues. This is where Magill's endotracheal technique has proved invaluable complement, particularly using the tube with an inflatable cuff, whi provides a gas-tight joint in the trachea, and so gives the anaesthetist complete con of respiration.

### RECENT ADVANCES IN ANAESTHESIA

Amongst other innovations, I should mention hypothermia, refrigeration or artific hibernation as it is variously called. For many years now there has been a desulto practice of local refrigeration, usually for amputation of leg in cases of gangre Nowadays, I would say that it is chiefly used for the purpose of demonstrating t method as such, but cannot be described as a normal everyday practice.

Complete cooling of the whole body to temperatures several degrees below normal i practice beset with problems, and the knowledge of the profound physiological seque is still limited. Briefly, the cooled cells demand very much less oxygen for survival, a so, in certain cardiac and aortic operations, where the systemic circulation may stopped for short periods, this lessened demand for oxygen may be of value. I wo say that these particular cases are the only ones where this extremely complica technique may be justified.

Induced hypotension is another procedure which is of great value in certain select types of operation, for instance, where blood loss is likely to be excessive at the norm blood-pressure, or where slight oozing may obscure perfect vision as in the microsco stage of the fenestration operation. Arfonad, the drug which is most generally used f this purpose, is usually administered by a drip, and gives minute-to-minute control of the blood-pressure, very frequent readings of which have to be taken throughout the operation.

New drugs are constantly being introduced, and I have not attempted to cover the whole range of complementary cocktails composed of "tranquillizer" drugs such as pethidine, largactil and others, but I have endeavoured to give a brief outline of the drugs and methods which have become the accepted practice.

### TRAVELS AND ADVENTURES

May I now give one or two amusing episodes which I have experienced?

Many years ago, before the last war, I was asked by a young surgeon to anaesthetize a very important industrial magnate. The condition was acute appendicitis, and pre-medication was with morphia and atropine. Induction was nitrous oxide and ether, and operation and convalescence were uneventful. Years later, I went to live next door to this magnate; you will understand that this was before the days of the N.H.S.; I live in a much smaller house now. Speaking to him one day, over the fence, he reminded me of the occasion of his operation, and told me that his last recollection before losing consciousness was hearing the surgeon say: "Thank heavens, Brad, this case has come along, I haven't seen a private case for a fortnight."

One foggy night, during the war, Mr. Cooke asked me to go with him to a cottage hospital in the country to deal with an alleged acute abdomen. So I packed my apparatus into his car, and off we set. After a very difficult journey through the black-out in addition to the fog, we arrived at the hospital. Was Mr. Cooke pleased when he saw the patient and diagnosed the case as acute alcoholism? The doctor and matron were very apologetic and placated us with bacon and eggs. However, our adventures were not over, for on the return journey we had to pass an aerodrome and, to add insult to injury, we were stopped by the guards and police who loomed out of the fog and nearly shut us up in the guard-room. It appears they were on the look-out for a similar car to Mr. Cooke's, and they appeared to be particularly suspicious of my anaesthetic apparatus, which at that time was packed in a sinister-looking black wooden box. Fortunately for us, however, word had just come through to say that the wanted car had been apprehended.

In 1948, I was privileged, at the kind invitation of Mr. Tasker, to accompany his surgical club, the Surgical Travellers, on a visit to Amsterdam and Utrecht. I was very interested to see the anaesthetic set-up there, at that time. It appeared that the Emeritus Professor of Surgery, Professor Nordenbos had recently visited London and was intensely interested in the progress made in cardiac surgery, and was anxious that his department should also enter the field. However, he realized that anaesthesia in his country did not fulfil the exacting requirements of cardiac surgery. So he had got into conversation with the young lady who was anaesthetizing one of these cardiac cases, and found that her name was Mrs. Vermeulen-Cranch, and that her husband was a Dutchman, living in Amsterdam, where she was hoping to join him as accommodation, which was very scarce, could be found. Professor Nordenbos saw this was a golden opportunity and certainly let no grass grow under his feet. This was on a Friday, and on the following Monday Mrs. Vermeulen was in Amsterdam, installed in a flat and appointed to the staff of the hospital. Other Dutch surgeons visiting Amsterdam saw some of this epoch-making cardiac surgery, and realizing the important part that anaesthesia took, inquired if they could send their assistants along for a week or two, to learn a bit about it, having, you see, no conception of the implications and responsibilities of modern anaesthesia. Dr. Vermeulin rightly pointed out that this was quite impossible, but that, if the University would arrange for them to attend the University and Hospital for a period of two years on a full-time course, she would undertake their tuition. And, so when I was there in 1948, she had six Dutch graduates learning the specialty of anaesthesia. On visiting Utrecht, I found a young English anaesthetist there, Dr. Roberts, who also had six Dutch trainees. Previous to Dr.

Roberts's arrival, Mrs. Vermeulen used to travel to Utrecht as well, to administer and teach anaesthetics, but the work grew to such an extent that she could not cope with it all, and had to confine her activities to Amsterdam. At that time the Dutch were full of admiration for the wonderful English anaesthesia as they called it. I have no doubt that by now, they call it their Dutch anaesthesia.

You may have noticed I called this lady Mrs. Vermeulen and sometimes Mrs. Vermeulen-Cranch. Her maiden name was Miss Cranch, and she was a Cardiff graduate. It appears to be a custom in Holland for the wife to tack her maiden name on the end of her husband's surname.

### GROWTH OF ANAESTHETICS AS A SPECIALTY

Well, ladies and gentlemen, during the past thirty years, we have certainly advanced from the old rag-and-bottle methods, and although these various developments seemed, at the time, to appear at fairly leisurely intervals, now, in retrospect, the impression on my mind is kaleidoscopic, and it is quite certain that much of the major operative surgery of today could not be undertaken if surgery and anaesthesia had not advanced hand-in-hand.

In the early 'twenties, textbooks on anaesthesia were few in number; now we have an extensive library. The oldest Society is that of England, known as the Society of Anaesthetists. It began in 1893, and went on until 1908, when it became the Section of Anaesthesia of the R.S.M. It was about 1906 or 1907 that many medical bodies united to form the single R.S.M. The Society of Anaesthetists published *Transactions*, small booklets you could put in your pocket, between 1898 and 1908. The Association of Anaesthetists of Great Britain and Ireland was founded in 1932 with Dr. Harry Featherstone as the President. It publishes *Anaesthesia* which began publication in 1946. The American Society of Anaesthesiology was founded in Brooklyn, New York in 1905, and publishes *Anaesthesiology* which began in 1940. The *British Journal of Anaesthesia* started in 1923. There is a World Federation of Societies of Anaesthetists with a membership of forty-two national societies.

An important step in the development of anaesthesia was the creation of the Chair of Anaesthesia at Oxford in 1936. At this time, Lord Nuffield had offered £1¼ million for the endownment of a Medical School Trust, including the establishment of five Chairs including one in anaesthesia. He had just announced that he had decided to increase his endowment to a round £2 million. Some doubt was expressed whether anaesthesia was really a subject for a chair but Nuffield, who had personal experience of anaesthesia both skilfully and unskilfully administered, persisted in his viewpoint that it would benefit from a Professor who could devote himself to its advancement through teaching and research. Sir Farquhar Buzzard was then Regius Professor of Medicine, and I quote from Nuffield's biography. "He remembers walking up and down with Buzzard arguing, and finally making it clear that he felt so strongly about it that his backing of the whole scheme would be in doubt." In this way, Oxford got the first chair in anaesthetics in the British Empire: others have followed since then, and the speciality has benefited by its progress being sustained at the highest academic levels. How right Lord Nuffield was, and how fortunate in the election of the first holder of the Chair, Professor Macintosh, now Sir Robert Macintosh, who has proved himself a veritable ambassador of anaesthesia.

The D.A. of the R.C.S. was instituted in 1935. The Faculty of Anaesthetists of the R.C.S. was created in 1948 and the higher diploma of F.F.A.R.C.S. inaugurated, which has a primary and final examinations.

The modern anaesthetist must be a sound clinician, whose opinion is valued in assessing the anaesthetic risks of operation. He has to have a sound knowledge of the basic sciences, to have knowledge of a large number of pharmacological preparations, and to know how to use them with safety. He must have a working knowledge of the mechanics of the apparatus he uses, and a more intimate knowledge of the effect of gases and relative pressures and their effects on the chemistry of the blood and tissues.

He must have a useful pair of hands, for he has to use endoscopes with precision and without trauma; he has to be capable of setting up intravenous drips of various types with speed at short notice. Last but not least, he must have a kind and sympathetic approach to the patient.

We have been accused, ladies and gentlemen, of making anaesthesia a "closed shop". The sole object, I can assure you, in raising the standard of administration of anaesthetics, and this inevitably entails a long and arduous apprenticeship, is to ensure for you and yours and the public in general, safety and comfort during surgical operations, and conditions under which the surgeon is able to give of his best. I, personally, wish to banish the day when administration of an anaesthetic is a hazardous, uncharted adventure into the unknown, both for the patient and the doctor, but welcome the time when, should the emergency arise, we can be admitted to any hospital in the country, with complete confidence that the anaesthetic will be administered, not by anyone at hand willing to have a bash, but by a properly-trained anaesthetist, with all that this implies, and so truly uphold the motto of the Association of Anaesthetists, "In somno securitas".

